# Cultural modulation effects on the self-face advantage: Do Caucasians find their own faces faster than Chinese?

**DOI:** 10.1177/17470218221142158

**Published:** 2022-12-15

**Authors:** Jasmine KW Lee, Chantelle Gregson, Steve MJ Janssen, Alejandro J Estudillo

**Affiliations:** 1 University of Nottingham Malaysia, Semenyih, Malaysia; 2 Bournemouth University, Poole, UK

**Keywords:** Self-face advantage, visual search, self-concept, culture

## Abstract

The self-face advantage (SFA) is reflected through a faster recognition of a self-face compared with familiar and unfamiliar faces. Nevertheless, as Westerners and East Asians tend to present differences in self-concept styles, it is possible that the SFA is modulated by culture. The present study explored this possibility using a visual search task. British Caucasians and Malaysian Chinese participants were asked to search for frontal view images of self, friend, and unfamiliar faces among an array of unfamiliar faces. Regardless of race, participants were more accurate and faster in searching for the own face and friend’s face compared with an unfamiliar face, with no differences in the search between the own and friend’s face, and these findings could not be accounted by the cultural differences in self-concept (i.e., operationalised by scores from the Independent and Interdependent Self-Concept Scale and the Horizontal and Vertical Individualism and Collectivism Scale). Altogether our results suggest that culture does not modulate the SFA and that this effect is better explained by a familiar face advantage.

One’s own face is presumably one of the most distinctive physical features (Tsakiris, 2008) of an individual and is arguably a unique self-referential stimulus not shared with others (Devue & Brédart, 2008). Indeed, one’s own face is strongly tied to identity and self-consciousness (e.g., Estudillo & Bindemann, 2017b; McNeill, 1998) and the ability to recognise one’s own face helps to maintain a sense of self (Estudillo & Bindemann, 2016, 2017a; Platek et al., 2004). One’s own face thus holds a special meaning to humans and is asserted to be processed distinctively. Contrary to other faces, the own face is postulated to show processing differences, with stronger feature-based processing (e.g., Greenberg & Goshen-Gottstein, 2009) and different electrophysiological and Blood Oxygen Level Dependent (BOLD) responses (Alzueta et al., 2019; Devue & Brédart, 2011; Estudillo, 2017; Estudillo et al., 2018). All this evidence suggests that, in comparison to other people’s faces, one’s own face is represented robustly in the mind.

The own face receives attentional priority and is processed faster compared with other faces (for recent review, see Bortolon & Raffard, 2018). This self-face advantage (SFA) is reflected through individuals demonstrating faster recognition to a self-face than to a stranger’s or a familiar other face (e.g., Keyes & Brady, 2010; Tong & Nakayama, 1999). For instance, individuals tend to show a faster and more efficient processing for own faces than for other faces (e.g., Keenan et al., 2000; Tong & Nakayama, 1999), and this advantage persists even for inverted views of faces (Keyes & Brady, 2010). In a classic study, Tong and Nakayama (1999) asked their participants to search for their own face or a stranger’s face among different sets of foil faces. The results showed that, compared with an unfamiliar target face, the self-face was consistently detected faster among the distractors. Interestingly, this SFA was also evident after hundreds of presentations of the unfamiliar face and with different face orientations (i.e., inverted, three-quarter, and profile views). These results suggest that people possess a robust mental representation of their own face, which is flexible enough to be generalised to inverted and atypical views of the own face.

The study by Tong and Nakayama (1999), however, compared search times for a highly familiar face (i.e., the self-face) with an unfamiliar face. Thus, it is not clear whether their results reflect a SFA or a general robust representation for highly familiar faces (Estudillo, 2012). To control for possible familiarity effects, Keyes and Brady (2010) tested participants in a face identification task with the own face, a personally familiar face, and an unfamiliar face. Results showed that, when compared with friend and unfamiliar faces, participants are still faster and more accurate at identifying self-faces.

Furthermore, with a face–name interference paradigm study, Brédart et al. (2006) also showed the attention-grabbing property of one’s own face. The detection of a classmate’s name is strongly interfered by a flanking self-face compared with the reversed condition, suggesting that self-faces have a stronger tendency to capture attention and are harder to ignore. Existing evidence, however, suggests a rather inconclusive effect of the attentional capture properties of self-face (i.e., SFA). For instance, adopting a similar visual search paradigm to that of Tong and Nakayama (1999), Devue et al. (2009) examined if the prioritisation of self-faces among highly familiar and unfamiliar faces is a “truly bottom-up” process. To examine if people show a “bottom-up” prioritisation to their own face, observers were required to search for a particular mouth configuration (i.e., M or O) in different types of face displays (i.e., self, friend, or neutral), while ignoring the face identity. Thus, in this task and in contrast to previous studies, face identity was task irrelevant. In contrast to the findings of Tong and Nakayama (1999), the self and friend faces were detected at a similar rate and there was no difference in the interference caused by the self or friend’s face. In other words, when detecting the target, the presence of a friend’s face showed a similar effect to that of the presence of a self-face. With such results, Devue et al. (2009) concluded that the SFA is only evident when face identity is task relevant.

## Cultural modulation

Another factor that seems to play an important role in the SFA is culture. There is consistent evidence demonstrating a varying importance of the self-face across cultures. For instance, using a head orientation judgement task, Sui et al. (2009) showed that British participants responded faster and more accurately to their own face relative to a friend’s face. In contrast, such an advantage was not found in Chinese participants. Another study showed that Chinese participants displayed no or a weakened SFA in the presence of their supervisor, but this decrease was not observed in British participants (Liew et al., 2011).

In line with these studies, it has been shown that culture plays a key role in determining one’s self-concept, with distinct self-concept styles for East Asian and Western cultures (Liew et al., 2011). Self-concept is generally understood as the way in which people perceive and evaluate themselves (Markus & Kitayama, 1991). Individuals from Western cultures (e.g., White Americans) demonstrate an independent self-concept. In these cases, they tend to be more individualistic, and the self is generally perceived as an autonomous entity (Markus & Kitayama, 1991). East Asians (e.g., Chinese), on the other hand, tend to demonstrate an interdependent self-concept, in which they value the interconnectedness with others and the self is generally conceptualised in terms of its relationships with others and social contexts (Markus & Kitayama, 1991). It is suggested that individuals with independent self-concepts assign a greater social salience or positive associations to self-faces than those with interdependent self-concepts (e.g., Ma & Han, 2009). Thus, independent self-concepts should lead to stronger attentional bias to self-related stimuli, such as the self-face, and, consequently, to an advantage in the processing of self-faces (i.e., SFA). Conversely, as interdependent self-concepts value the interconnectedness with others, self-face might be as relevant as friend’s faces, which should diminish the SFA (Sui et al., 2009).

## The current study

Hence, this study was conducted to examine cultural modulation effects on the SFA with a visual search paradigm. Although both Tong and Nakayama (1999) and Devue et al. (2009) used a similar visual search paradigm, they reported contradictory results: an SFA was reported in the former but not in the latter study. Discrepancies in the task might explain this difference. Tong and Nakayama (1999) did not control for possible familiarity effects, whereas face identity was task irrelevant in Devue et al. (2009). To control for familiarity effects but otherwise to replicate the design of Tong and Nakayama (1999) as closely as possible, personally familiar faces (e.g., a friend’s face) will be included and face identity will be task relevant in our study.

For this study, we hypothesised that the SFA might be modulated by the cultural differences in the self-concepts of participants, where we expect people with independent selves (i.e., British Caucasians) to show a robust SFA and people with interdependent selves (i.e., Chinese Malaysians) to show a weakened SFA. To test this hypothesis, this study included British Caucasians and Malaysian Chinese and compared their search times and accuracy for frontal view images of self, friend, and unfamiliar faces among an array of unfamiliar distractor faces. Specifically, we anticipated that Caucasians would demonstrate faster reaction times (RT) and higher accuracy when searching for their own face compared with their friend’s face as independent selves would show stronger attention to self-faces. Conversely, as the interdependent selves place more emphasis on the interconnectedness between self and others, we anticipated that when searching for their own face and friend’s face, Chinese participants would show a smaller difference in terms of the RT and accuracy than Caucasian participants.

As both the self-face and the friend’s face are—due to extensive exposure—highly overlearned faces, both faces should show a processing advantage compared with the unfamiliar face in both race groups. For example, di Oleggio Castello et al. (2017) observed that Caucasian individuals demonstrated a shorter searching time for a familiar target compared with an unfamiliar target in a visual search task. A similar pattern has also been reported for Asian participants (Zhang & Zhou, 2019). Therefore, we expected that, regardless of the race of participants, the self-face and the friend’s face will have a familiarity advantage compared with the unfamiliar face. Specifically, we anticipate that both Caucasians and Chinese would demonstrate shorter search times and higher accuracy for familiar faces compared with unfamiliar faces.

In addition, we also explored whether an SFA can be explained by the differences in self-construal of the participants, regardless of their race. The Independent and Interdependent Self-Construal Scale (SCS; Singelis, 1994) was used to assess the independent and interdependent self-concepts among participants. For this explorative analysis, we expected that, regardless of the race of the participants, individuals with a higher score on the independent self-construal subscale would show a stronger SFA whereas individuals with a higher score on the interdependent self-construal subscale would show a weaker SFA.

Although this questionnaire has been widely used in other face processing studies concerning cultural modulation effects (e.g., Ma & Han, 2010; Sui et al., 2012), it has low internal consistency scores that range from high .60s to middle .70s (Singelis, 1994). To address this reliability issue, we included another scale, the Horizontal and Vertical Individualism and Collectivism Scale (HVIC; Triandis & Gelfand, 1998) that measures multidimensional construct of individualistic and collectivism by characterising it into horizontal (highlights equality) and vertical (highlights hierarchy) social relationship terms: namely, Horizontal Collectivism (HC), Horizontal Individualism (HI), Vertical Collectivism (VC), and Vertical Individualism (VI).

Horizontal patterns of social relationship assume oneself is similar to other selves (i.e., a preference for equality), whereas vertical patterns comprise of hierarchy and involvement of authority, wherein each self is distinct from other selves (i.e., a preference for hierarchy; see Triandis & Gelfand, 1998). Specifically, in cases of HI, individuals are more self-reliant and aspire to be unique from others, yet these individuals are less interested in acquiring a high social status. In cases of VI, individuals tend to care for acquiring status through individual competition with others. HC corresponds to individuals perceiving themselves similar to others and they give emphasis to interconnectedness and sharing common goals with groups. However, they do not yield easily to authority. Finally, VC individuals are typically characterised with their willingness to sacrifice own ideals for the benefit of the in-group goals. For this analysis, we expected that, regardless of the race of participants, individuals with a higher score on both HI and VI would show a stronger SFA whereas individuals with a higher score on both HC and VC would show a weaker SFA.

## Method

### Participants

Fifty-six Malaysian Chinese and 56 British Caucasian students were recruited from the University of Nottingham Malaysia and Bournemouth University, respectively. A power analysis performed in G*Power 3.1 (Faul et al., 2007) with the smallest effect size of interest (Lakens et al., 2018) of 0.10 and an alpha of .05 gives a required total sample size of 112 participants (56 participants for each group) to achieve 80% power in a mixed-design analysis of variance (ANOVA).

Participants were recruited in pairs matched by age, gender, and race, so that each served as the friend for the other participant. The age range allowed for matching is up to 3 years. Participants were either awarded with course credits or compensated financially for their participation. Ethics approval for this study was obtained from the Science and Engineering Ethics Committee of the University of Nottingham Malaysia and the Ethics Committee of Bournemouth University.

### Materials

#### Image collection

**Figure 1.**
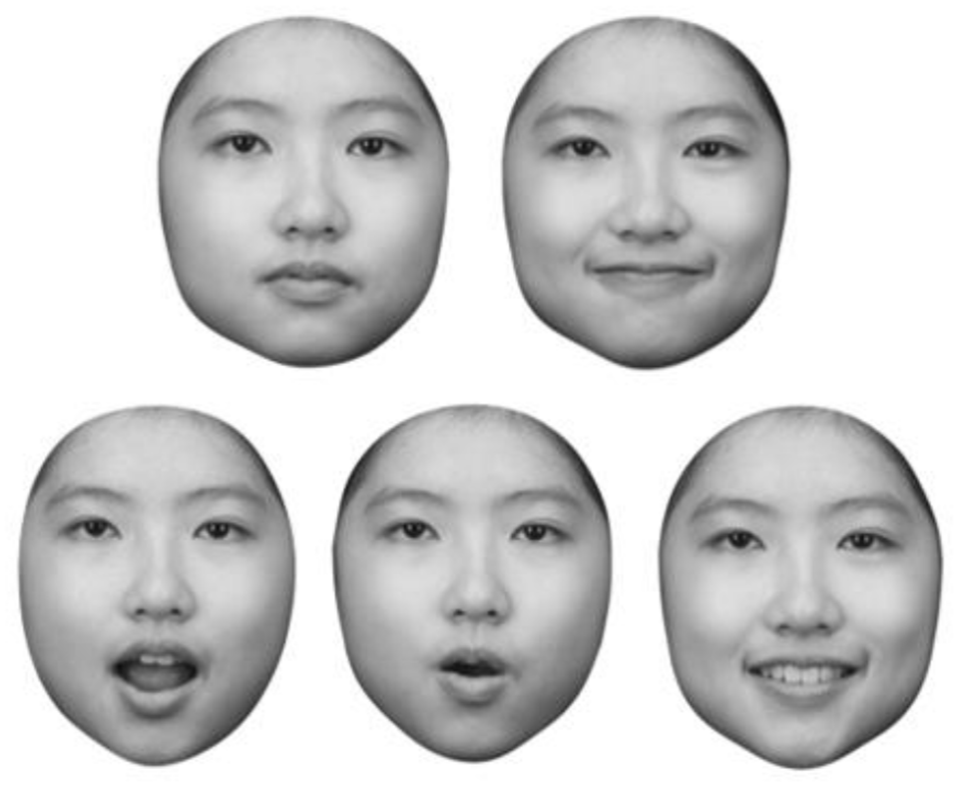
Example of face stimuli. The five different images for each identity that were presented throughout the study. From left to right, top row: neutral, happy. From left to right, bottom row: “A,” “O,” and “E.”

Photograph stimuli (self-face and friend face) were individually tailored to each participant. Each participant was photographed under similar conditions (i.e., constant lighting), in a frontal position while assuming a neutral and a happy expression and while articulating three different speech sounds (e.g., A, O, and E; see Figure 1). Different images were used for each identity to reduce image-specific learning. All five different images were used as “self-face” for the participant themselves and as “friend’s face” for their friend respectively. Twenty-eight separate individuals: 14 Caucasians (7 males and 7 females) and 14 Chinese (7 males and 7 females) matched in age were photographed under same conditions to be used as unfamiliar targets and distractor faces. All images were collected and processed at least 1 week prior to the experimental session.

#### Image processing

Using Photoshop, all photographs were rotated to ensure eyes are collinear and were cropped to 113 × 126 pixels, corresponding to an approximate visual angle of 2.9° × 3.4° at a viewing distance of 70 cm. All photographs were cropped based on their individual contours and external features (i.e., hairs and ears were removed). All face images were also converted to greyscale. These transformations would minimise differences in non-facial cues.

“Self-face” images were presented in a mirror-reversed orientation (i.e., the view in which people generally view their own face), whereas the “friend” and “unfamiliar” images were presented in normal orientation. Each participant’s stimuli set consisted of four sets of images: one target self-face set (with five different images), one target friend face set (with five different images), one target unfamiliar face set (with five different images), and six distractor faces sets (each with five different images). Figure 1 shows an example of face stimuli that were presented in the study.

After the main experiment, a subsequent study was conducted to assess the similarity of the face stimuli across target conditions and race groups. This assessment could only be conducted afterwards because the self-face and friend face stimuli were not available before the visual search task had been completed. Ten independent raters from each race group (who did not participate in the main experiment) were asked to rate how much each of the own-race faces used in the different conditions of the visual search task (self, friend, unfamiliar, and distractor) stands out. Specifically, they were asked to rate “how likely will this face stand out in a crowd?” on a 5-point Likert-type scale, with 1 = *not at all likely* and 5 = *extremely likely*. Faces were presented individually in the centre of the screen until participants respond.

With these scores, a “stand-out” score for each face condition compared with the mean stand-out score for the distractor faces was calculated. Specifically, we calculated for each participant how much the familiar faces (i.e., self-face or friend face) stood out compared with distractor faces (SF or FF–DF), and how much the unfamiliar face stood out compared with the distractor faces (UF–DF). Because participants were recruited in pairs, there would only be one set of familiar faces, as the face of each participant would have two roles (self-face and friend face). Finally, two independent-samples *t*-tests were conducted with the two stand-out scores as the dependent variables and race group as the independent variable. If any of these *t*-tests showed differences across race groups, these stand-out scores would be included as covariates in the analyses of the visual search task.

The stand-out scores between familiar faces (SF or FF) and distractor faces did not differ significantly across Chinese and Caucasian participants, *t*(18) = 1.09, *p* = .291. Similarly, the stand-out scores between unfamiliar and distractor faces also did not differ significantly across Chinese and Caucasian participants, *t*(18) = −0.84, *p* = .414.

#### Independent and interdependent SCS

This scale consists of 30 statements (15 independent and 15 interdependent items) that measure the two distinct dimensions of self-construal (Singelis, 1994). Participants were required to indicate their agreement with the statements on a 7-point Likert-type scale, ranging from 1 = *strongly disagree* to 7 = *strongly agree*. Using Cronbach’s alpha, previous research reported that the internal consistency of the interdependent self-construal subscale was .59, whereas the internal consistency of the independent self-construal subscale was .60 (Kim et al., 1994).

#### Horizontal and Vertical Individualism and Collectivism Scale

This scale consists of 16 items that measure four different dimensions of collectivism and individualism, namely horizontal (H) and vertical (V) individualism (I) and collectivism (C), making up HI, VI, HC, and VC. Each dimension consists of four items. For instance, an item from HI dimension is “I rely on myself most of the time; I rarely rely on others”; an example item from VI is “It is important that I do my job better than others”; an item from HC is “I feel good when I cooperate with others”; and an item from VC is “It is important to me that I respect the decisions made by my groups.” Participants were required to indicate their agreement with the statements on a 9-point Likert-type scale ranging from 1 = *never or definitely no* to 9 = *always or definitely yes*. Each dimension’s items are summed up separately to create a HI, HC, VI, and VC score.

### Procedure

This study used a mixed design with one between-subjects variable (race: Chinese or Caucasian) and two within-subjects variables (target identity: self, friend, and unfamiliar; and target presence: present or absent). A total of six blocks with each target identity condition was presented twice. The presentation of blocks was counterbalanced for target identity (i.e., self, friend, or unfamiliar face) where target identity changed from one block to the next.

Each block consisted of a total of 80 trials wherein target faces appeared in only 50% of the trials (i.e., target present condition): 40 (5 different target images × 8 repetitions). The remaining 50% of the trials consisted of display of only unfamiliar distractor faces (i.e., target absent condition). The order of trials within each block was randomised as well. The distractor faces were randomly selected among the set of six distractors with no two identical faces presented within the same trial. For each trial, participants’ set of stimuli (self, friend, unfamiliar, and distractor faces) would always consist of the same emotional expression, race, and gender. At the start of the study, participants performed a familiarisation phase: 36 practice trials with the same unfamiliar target during the practice trials as during the subsequent test trials.

**Figure 2.**
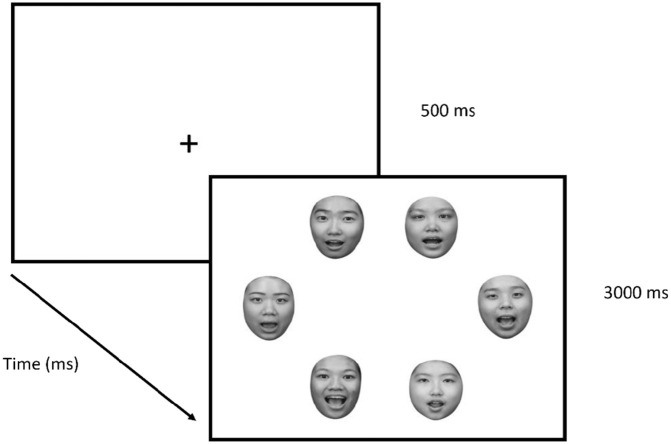
The experimental paradigm. On each trial, a central fixation cross was presented for 500 ms, followed by an array of six faces for a maximum of 3,000 ms.

During the experiment, participants were seated 70 cm from the screen. The screen measured horizontally 51 cm and vertically 28.5 cm. Participants were then instructed to search for a given target identity among an array of distractor faces. At the start of each block, participants were cued with a target image (i.e., self-face, friend face, or unfamiliar face). With a key press by the participants, each trial was initiated with a central fixation cross appearing for 500 ms. Participants were asked to fixate the cross until an array of six faces is presented. All face stimuli (i.e., target face and distractor faces) were randomly positioned to one of the six possible locations to form a hexagon around a fixation cross subtending to a visual angle of 10.1° × 7.7° (see Figure 2). The display remained on screen for 3 s or until participants made a response. The target face was present in 50% of the trials, and to respond, participants pressed the “/” key when the target was present and the “z” key when the target was absent. Participants were asked to respond as quickly and as accurately as possible, and visual feedback was provided when the response was incorrect or when participants did not respond within 3 s.

Participants were also asked to complete the SCS and HVIC questionnaires. They were asked to answer these questionnaires prior to performing the visual search task. The study took about 40 min to complete.

### Data analyses

As processing efficiency (i.e., RT is often used as a criterion to determine an SFA) is our main interest, data analysis was performed on the median RT of correct responses. The median of RT was used instead of mean RT to remove the influence of extreme values. Accuracy was recorded and used an outcome variable as well.

## Results

To test for the effects of cultural modulation of the SFA, in the first part of the analysis, participants were grouped according to their ethnicity. Two 2 (*Race: Chinese or Caucasian*) × 3 (*Target Identity: Self (SF), Friend (FF), or Unfamiliar (UF))* × 2 (*Target Presence: Present or Absent*) mixed-design ANOVAs were conducted on the median RTs and search accuracy for correct responses, with race as the between-subject variable and target identity and target presence as the within-subject variables.

### Median RT

**Figure 3.**
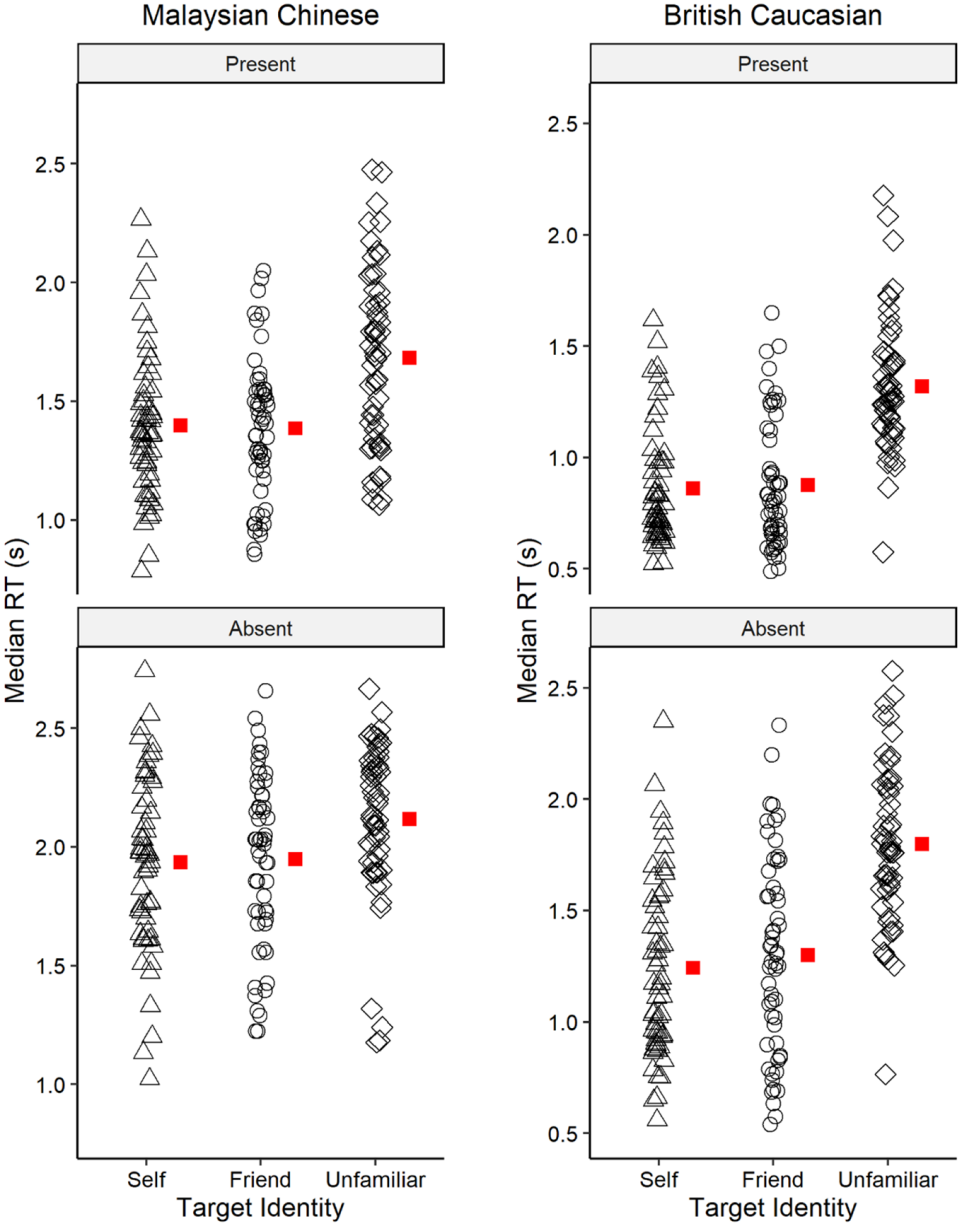
The search time of Malaysian Chinese and British Caucasian participants. Median RT per participant for self-face, friend’s face, and unfamiliar faces across present and absent trials. Red square denotes the group mean.

Figure 3 shows the median RT for each race across different identity in target present and absent trials, respectively. The analysis revealed a significant main effect for race, *F*(1, 110) = 118.27, *p* < .001, η_p_^2^ = .518, with shorter search times for the British Caucasians (*M* = 1.23, *SD* = 0.47) than for the Malaysian Chinese (*M* = 1.75, *SD* = 0.44) participants. In addition, a significant main effect of target identity was reported, *F*(1.68, 185.48) = 86.23, *p* < .001, η_p_^2^ = .439 (Huynh–Feldt corrected). Holm–Bonferroni post hoc comparisons indicated that participants searched SF (*M* = 1.36, *SD* = 0.51) faster than UF (*M* = 1.73, *SD* = 0.44; *t* = −11.80, *p* < .001, *d* = −1.12), and searched FF (*M* = 1.39, *SD* = 0.54) faster than UF (*t* = −10.70, *p* < .001, *d* = −1.02), but there was no significant difference in the search time for the SF and FF (*t* = −1.10, *p* = .273, *d* = −0.10). The analysis also revealed a significant main effect of target presence, *F*(1, 110) = 706.48, *p* < .001, η_p_^2^ = .865, with participants responding faster in the present trials (*M* = 1.25, *SD* = 0.42) compared with the absent trials (*M* = 1.73, *SD* = 0.51).

The analysis further showed a significant interaction effect between race and identity, *F*(1.69, 185.48) = 12.43, *p* < .001, η_p_^2^ = .102 (Huynh–Feldt corrected), and between race and target presence, *F*(1, 110) = 8.12, *p* = .005, *η*_p_^2^ = .069. Both two-way interactions were qualified by a significant three-way interaction between race, identity, and target presence, *F*(1.67, 183.80) = 9.68, *p* < .001, η_p_^2^ = .081 (Huynh–Feldt corrected).

To understand these interactions further, we conducted simple main effect analyses for each level of race. An ANOVA on the median RT for Malaysian Chinese showed a significant main effect of identity, *F*(1.51, 83.29) = 11.47, *p* < .001, η_p_^2^ = .173 (Huynh–Feldt corrected), a significant main effect of target presence, *F*(1, 55) = 439.23, *p* < .001, η_p_^2^ = .889, and a significant interaction effect between identity and target presence, *F*(1.50, 82.46) = 7.96, *p* = .002, η_p_^2^ = .126 (Huynh–Feldt corrected). Holm–Bonferroni post hoc comparisons revealed that in target present trials, participants search the SF (*t* = −4.56, *p* < .001, *d* = −0.61) and FF (*t* = −4.73, *p* < .001, *d* = −0.63) faster than UF, but there were no significant differences in the search time for SF and FF (*t* = 0.18, *p* = .849, *d* = 0.02). In the target absent trials, participants searched the SF faster than UF (*t* = −3.44, *p* = .002, *d* = −0.46) but there were no significant differences in the search time between SF and FF (*t* = −1.34, *p* = .182, *d* = −0.18) and FF and UF (*t* = −2.09, *p* = .077, *d* = −0.28).

Next, an ANOVA on the median RT for British Caucasians also revealed a significant main effect of identity, *F*(2, 110) = 149.67, *p* < .001, η_p_^2^ = .731 (Huynh–Feldt corrected), a significant main effect for target presence, *F*(1, 55) = 277.65, *p* < .001, η_p_^2^ = .835, and a significant interaction between identity and target presence, *F*(2, 110) = 3.78, *p* = .026, η_p_^2^ = .064. Holm–Bonferroni post hoc comparisons revealed in target present trials, SF (*t* = −12.33, *p* < .001, *d* = −2.08) and FF (*t* = −11.90, *p* < .001, *d* = −2.01) was searched faster than UF, but there were no significant differences in the search time for SF and FF (*t* = −0.43, *p* = 1.00, *d* = −0.07); in the target absent trials, SF (*t* = −12.76, *p* < .001, *d* = −1.71) and FF (*t* = −11.42, *p* < .001, *d* = −1.53) was also searched faster than UF, whereas there were no significant differences in the search time between SF and FF (*t* = −1.34, *p* = .353, *d* = −0.18).

In summary, we hypothesised that British Caucasians would search SF faster than FF, demonstrating a robust SFA, but Malaysian Chinese would show a smaller SFA than British Caucasians. However, our results did not support this hypothesis. We also expected that participants would search familiar faces (SF and FF) faster than UF, regardless of participants’ race. Our results supported this hypothesis, as both groups of participants were faster in searching for SF and FF than UF, but no differences were found between SF and SF. Finally, our findings also revealed that compared with Malaysian Chinese, British Caucasians were overall faster in searching for faces, regardless of their identity.

### Search accuracy

**Figure 4.**
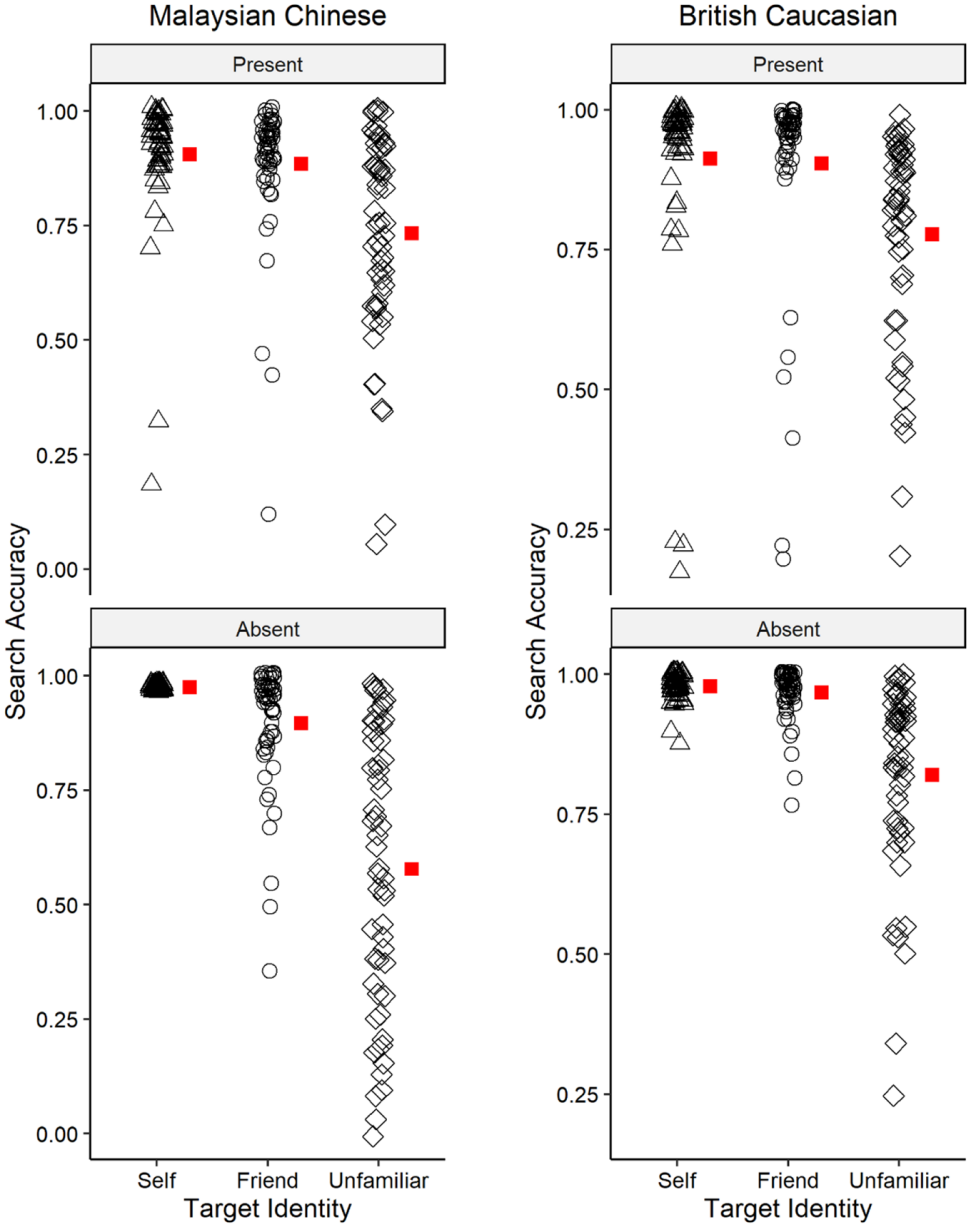
The search accuracy of Malaysian Chinese and British Caucasian participants. The mean accuracy scores per participant for self-face, friend’s face, and unfamiliar face across present and absent trials. Red square denotes the group mean.

Figure 4 shows the search accuracy for each race across different identity in target present and absent trials, respectively. The analysis revealed a significant main effect for race, *F*(1, 110) = 18.26, *p* < .001, η_p_^2^ = .142, with a higher accuracy for British Caucasians (*M* = 0.893, *SD* = 0.16) than Malaysian Chinese (*M* = 0.820, *SD* = 0.23) participants. A significant main effect of target identity was also found, *F*(1.34, 144.77) = 108.64, *p* < .001, η_p_^2^ = .497 (Huynh–Feldt corrected). Holm–Bonferroni post hoc comparisons indicated that participants performed significantly better when searching for the SF (*M* = 0.930, *SD* = 0.13; *t* = 13.26, *p* < .001, *d* = 1.26) and FF (*M* = 0.913, *SD* = 0.14; *t* = 12.14, *p* < .001, *d* = 1.15) than for the UF (*M* = 0.727, *SD* = 0.24), but there was no significant difference between the SF and FF (*t* = 1.12, *p* = .265, *d* = 0.11).

The analysis further showed a significant interaction effect between race and target identity, *F*(1.34, 147.17) = 8.13, *p* = .002, η_p_^2^ = .069, between race and target presence, *F*(1, 110) = 8.52, *p* = .004, η_p_^2^ = .072, and between identity and target presence, *F*(1.34, 147.89) = 15.05, *p* < .001, η_p_^2^ = .120 (Huynh–Feldt corrected). All two-way interactions were qualified by a significant three-way interaction between race, identity, and target presence, *F*(1.34, 147.89) = 8.88, *p* = .001, η_p_^2^ = .075 (Huynh–Feldt corrected).

To understand these interactions further, we conducted simple main effects analysis for each level of race. An ANOVA on the accuracy data for Malaysian Chinese showed a significant main effect of identity, *F*(1.35, 74.16) = 59.36, *p* < .001, η_p_^2^ = .519 (Huynh–Feldt corrected). The analysis also revealed that identity interacted significantly with target presence, *F*(1.20, 65.84) = 15.28, *p* < .001, η_p_^2^ = .217 (Huynh–Feldt corrected). Holm–Bonferroni post hoc comparisons revealed that in target present trials, participants searched the SF (*t* = 7.26, *p* < .001, *d* = 0.97) and FF (*t* = 6.39, *p* < .001, *d* = 0.85) better than UF, whereas there were no significant differences in the search accuracy for SF and FF (*t* = 0.87, *p* = 1.00, *d* = 0.17); in the target absent trials, participants searched the SF (*t* = 9.10, *p* < .001, *d* = 1.22) and FF (*t* = 8.34, *p* < .001, *d* = 1.11) better than UF, but there were no significant differences in the search accuracy for SF and FF (*t* = 0.75, *p* = 1.00, *d* = 0.10). The analysis revealed no main effect of target presence, *F*(1, 55) = 2.90, *p* = .094, η_p_^2^ = .050.

Next, an ANOVA on the accuracy data for British Caucasians revealed a significant main effect of identity, *F*(1.30, 71.20) = 55.79, *p* < .001, η_p_^2^ = .503 (Huynh–Feldt corrected). Holm–Bonferroni post hoc comparisons showed that participants performed better when searching for the SF (*t* = 9.43, *p* < .001, *d* = 1.26) and FF (*t* = 8.81, *p* < .001, *d* = 1.18) compared with the UF, but there were no significant differences between the search accuracy for the SF and FF (*t* = 0.62, *p* = .538, *d* = 0.08). The analysis further revealed a significant main effect for target presence, *F*(1, 55) = 6.01, *p* = .017, η_p_^2^ = .099, with a higher search accuracy for absent trials compared with present trials. The analysis also revealed no significant interaction between target identity and target presence, *F*(1.82, 100.29) = 0.89, *p* = .408, η_p_^2^ = .016.

To summarise, we hypothesised that British Caucasians would search SF better than FF demonstrating a robust SFA, and that this SFA would be smaller in Malaysian Chinese participants. Similar to our RT analysis, accuracy results did not support this hypothesis. We also hypothesised that participants, regardless of their race group, would search familiar faces (SF and FF) more accurately than UF. Our results supported this hypothesis. Finally, our findings showed that compared with Malaysian Chinese, British Caucasians were overall more accurate in searching for faces, regardless of the identity.

### SCS Questionnaire Analyses

To examine whether the SFA reported can be significantly predicted by the self-construal, regardless of the race of participants, six two-step hierarchical regressions were conducted with the difference in search accuracy or median RT between two target conditions: SF–FF, SF–UF, or FF–UF as the criterion variable. Race of participants was entered in the first step of the regression, whereas self-construal (i.e., difference between the scores on the two subscales of the SCS questionnaire) was entered in the second step. Table 1 shows the descriptive statistics for scores on SCS questionnaire and HCIV questionnaire, whereas the regression statistics for the median RT and accuracy are reported in Tables 2 and Table 3, respectively.

**Table 1.** Mean scores for the self-construal scale and for the horizontal and vertical individualism and collectivism scale reported by Malaysian Chinese and British Caucasian participants (*N* = 112).

Questionnaire measure	Malaysian Chinese	British Caucasians
Independence (IND)	70.59 (8.45)	70.20 (9.80)
Interdependence (INT)	73.88 (7.68)	71.38 (7.84)
Horizontal Individualism (HI)	27.20 (4.61)	27.07 (4.55)
Vertical Individualism (VI)	21.82 (5.38)	19.71 (5.82)
Horizontal Collectivism (HC)	27.80 (4.36)	27.89 (4.02)
Vertical Collectivism (VC)	27.11 (4.98)	24.59 (4.64)

Numbers in parentheses are *SD*s.

**Table 2.** Summary of hierarchical regression for variables predicting the SFA effect in search time.

	Variable	*B*	*SE B*	β	*T*	*R*	*R* ^2^	*∆R* ^2^
SF–FF	Step 1					.016	0	0
	Race	.008	.050	.016	.167			
	Step 2					.114	.013	.013
	Race	.014	.050	.026	.271			
	Self-construal	−.038	.032	−.113	−1.19			
SF–UF	Step 1					.430	.185	.185
	Race	−.351	.070	−.430	−5.00***			
	Step 2					.431	.186	.001
	Race	−.349	.071	−.428	−4.93***			
	Self-construal	−.015	.044	−.029	−.330			
FF–UF	Step 1					.443	.196	.196
	Race	−.359	.069	−.443	−5.18***			
	Step 2					.445	.198	.002
	Race	−.363	.070	−.447	−5.19***			
	Self-construal	.023	.044	.045	.521			

SFA: self-face advantage; SF: self; FF: friend; UF: unfamiliar.

*N* = 112; *p < .05, **p < .01, ***p < .001.

**Table 3.** Summary of hierarchical regression for variables predicting the SFA effect in search accuracy.

	Variable	*B*	*SE B*	Β	*T*	*R*	*R* ^2^	*∆R* ^2^
SF–FF	Step 1					.090	.008	.008
	Race	−.016	.016	−.090	−.946			
	Step 2					.114	.013	.005
	Race	−.017	.017	−.096	−1.00			
	Self-construal	.008	.010	.070	.730			
SF–UF	Step 1					.282	.079	.079
	Race	−.113	.037	−.282	−3.08**			
	Step 2					.292	.085	.006
	Race	−.111	.037	−.275	−2.99**			
	Self-construal	−.019	.023	−.077	−.839			
FF–UF	Step 1					.262	.069	.069
	Race	−.098	.034	−.262	−2.85**			
	Step 2					.287	.082	.013
	Race	−.094	.034	−.252	−2.74**			
	Self-construal	−.027	.022	−.116	−1.26			

SFA: self-face advantage; SF: self; FF: friend; UF: unfamiliar.

*N* = 112; *p < .05, **p < .01, ***p < .001.

#### Median RT

The hierarchical regression analysis revealed that race contributed significantly to the differences in the search time between SF and UF, *F*(1, 110) = 25.01, *p* < .001, and accounted for 18.5% of the variation of the differences whereas self-construal did not significantly predict the differences, *F*(1, 109) = 0.11, *p* = .742. Another hierarchical regression analysis revealed that race contributed significantly to the differences in the search time between FF and UF, *F*(1, 110) = 26.81, *p* < .001, and accounted for 19.6% of the variation in the search accuracy of the differences whereas self-construal did not significantly predict the differences, *F*(1, 109) = 0.27, *p* = .603. Finally, neither race, *F*(1, 110) = 0.03, *p* = .868, nor self-construal, *F*(1, 109) = 1.41, *p* = .237, contributed significantly to the differences between the search time of SF and FF.

#### Search accuracy

The hierarchical regression analysis revealed that race contributed significantly to the differences in the search accuracy between SF and UF, *F*(1, 110) = 9.47, *p* = .003, and accounted for 7.9% of the variation of the differences whereas self-construal did not significantly predict the differences, *F*(1, 109) = 0.70, *p* = .403. Another hierarchical regression analysis revealed that race contributed significantly to the differences in the search accuracy between FF and UF, *F*(1, 110) = 8.14, *p* = .005, and accounted for 6.9% of the variation in the search accuracy of the differences whereas self-construal did not significantly predict the differences, *F*(1, 109) = 1.58, *p* = .212. Finally, neither race, *F*(1, 110) = 0.89, *p* = .346, nor self-construal, *F*(1, 109) = 0.53, *p* = .467, contributed significantly to the differences between the search accuracy of SF and FF.

We hypothesised that, regardless of the race of the participants, individuals with higher scores on the independent self-construal subscale would show a stronger SFA whereas individuals with a higher score on the interdependent self-construal subscale would show a weaker SFA. However, contradicting the hypotheses, these results suggest that, for both the search time and search accuracy, the reported SFA relative to UF can be explained by participants’ race but not by participants’ self-construal as measured by the level of interdependence and independence on the SCS questionnaire.

### HCIV questionnaire analyses

Finally, due to the low internal consistency of the SCS questionnaire, an additional HCIV questionnaire was administered to provide support to the scores from SCS questionnaire. For this analysis, six two-step hierarchical regressions were conducted to examine whether the SFA effect reported can be significantly predicted by the level of individualism and collectivism of participants. The difference in search accuracy and median RT between of two target conditions: SF–FF, SF–UF, or FF–UF were entered as the criterion variable. Race of participants was entered in the first step of the regression, whereas HI scores, VI scores, HC scores, and VC scores were entered in the second step. The regression statistics for the median RT and search accuracy are reported in Tables 4 and 5, respectively.

**Table 4.** Summary of hierarchical regression for variables predicting the SFA effect in search time.

	Variable	*B*	*SE B*	Β	*T*	*R*	*R* ^2^	*∆R* ^2^
SF–FF	Step 1					.016	0	0
	Race	.008	.050	.016	.167			
	Step 2					.101	.010	.010
	Race	.020	.054	.039	.381			
	HI scores	−.002	.006	−.032	−.305			
	VI scores	.005	.005	.101	.933			
	HC scores	−.002	.007	−.038	−.367			
	VC scores	.001	.006	.016	.146			
SF–UF	Step 1					.430	.185	.185
	Race	−.351	.071	−.430	−5.00***			
	Step 2					.433	.187	.002
	Race	−.351	.075	−.431	−4.68***			
	HI scores	−.000	.009	−.001	−.007			
	VI scores	.003	.007	.039	.401			
	HC scores	−.001	.009	−.007	−.072			
	VC scores	−.002	.008	−.029	−.294			
FF–UF	Step 1					.444	.196	.196
	Race	−.359	.069	−.443	−5.18***			
	Step 2					.445	.198	.002
	Race	−.372	.074	−.458	−5.00***			
	HI scores	.002	.008	.020	.213			
	VI scores	−.002	.007	−.026	−.267			
	HC scores	.002	.009	.018	.191			
	VC scores	−.003	.008	−.040	−.403			

SFA: self-face advantage; SF: self; FF: friend; UF: unfamiliar; HI: horizontal individualism; VI: vertical individualism; HC: horizontal collectivism; VC: vertical collectivism.

*N* = 112; *p < .05, **p < .01, ***p < .001.

**Table 5.** Summary of hierarchical regression for variables predicting the SFA effect in search accuracy.

	Variable	*B*	*SE B*	Β	*T*	*R*	*R* ^2^	*∆R* ^2^
SF–FF	Step 1					.090	.008	.008
	Race	−.016	.016	−.090	−.946			
	Step 2					.247	.061	.053
	Race	−.027	.017	−.158	−1.59			
	HI scores	.000	.002	−.019	−.190			
	VI scores	−.002	.002	−.152	−1.44			
	HC scores	.000	.002	.016	.161			
	VC scores	−.003	.002	−.152	−1.42			
SF–UF	Step 1					.282	.079	.079
	Race	−.113	.037	−.282	−3.08**			
	Step 2					.326	.106	.027
	Race	−.112	.039	−.277	−2.87**			
	HI scores	−.003	.004	−.064	−.650			
	VI scores	−.003	.004	−.074	−.721			
	HC scores	−.006	.005	−.129	−1.31			
	VC scores	.003	.004	.068	.652			
FF–UF	Step 1					.262	.069	.069
	Race	−.098	.034	−.262	−2.85*			
	Step 2					.314	.099	.030
	Race	−.084	.036	−.227	−2.34*			
	HI scores	−.003	.004	−.061	−.609			
	VI scores	.000	.003	−.009	−.092			
	HC scores	−.007	.004	−.147	−1.48			
	VC scores	.005	.004	.144	1.37			

SFA: self-face advantage; SF: self; FF: friend; UF: unfamiliar; HI: horizontal individualism; VI: vertical individualism; HC: horizontal collectivism; VC: vertical collectivism.

*N* = 112; *p < .05, **p < .01, ***p < .001.

#### Median RT

The hierarchical regression analysis revealed that race had contributed significantly to the differences in the search time between SF and UF, *F*(1, 110) = 25.01, *p* < .001, and accounted for 18.5% of the variation of the differences whereas individualism and collectivism did not significantly predict the differences, *F*(4, 106) = 0.07, *p* = .992. Another hierarchical regression analysis revealed that race contributed significantly to the differences in the search time between FF and UF, *F*(1, 110) = 26.81, *p* < .001, and accounted for 19.6% of the variation in the difference between the search times whereas individualism and collectivism did not significantly predict the differences, *F*(4, 106) = 0.08, *p* = .990. Finally, neither race, *F*(1, 110) = 0.03, *p* = .868, nor the levels of individualism and collectivism of participants, *F*(4, 106) = 0.27, *p* = .900, contributed significantly to the differences between the search time of SF and FF.

#### Search accuracy

The hierarchical regression analysis revealed that race had contributed significantly to the differences in the search accuracy between SF and UF, *F*(1, 110) = 9.47, *p* = .003, and accounted for 7.9% of the variation of the differences whereas individualism and collectivism did not significantly predict the differences, *F*(4, 106) = 0.81, *p* = .524. Another hierarchical regression analysis revealed that race contributed significantly to the differences in the search accuracy between FF and UF, *F*(1, 110) = 8.14, *p* = .005, and accounted for 6.9% of the variation in the search accuracy of the differences whereas individualism and collectivism did not significantly predict the differences, *F*(4, 106) = 0.88, *p* = .480. Finally, neither race, *F*(1, 110) = 0.90, *p* = .346, nor the level of individualism and collectivism of participants, *F*(4, 106) = 1.49, *p* = .209, contributed significantly to the differences between the search accuracy of SF and FF.

We expected that, regardless of the race of the participants, individuals with a higher score on both HI and VI would show a stronger SFA whereas individuals with a higher score on both HC and VC would show a weaker SFA. Contradicting the hypotheses, these results suggest that, for both the search time and search accuracy, the reported SFA relative to UF can be explained by participants’ race but not by participants’ self-construal as measured by the level of individualism and collectivism in the HCIV questionnaire.

## Discussion

The aim of the current study was to identify the differences in the cultural background as a modulating factor of the SFA effect. We hypothesised that the SFA would be modulated by the cultural differences in the self-concepts of participants. Specifically, due to the cultural differences in the emphasis on the independent and interdependent self, we predicted that the SFA effect relative to a friend’s face would be larger in British Caucasians compared with Malaysian Chinese participants (i.e., SF–FF) whereas both race groups would show a comparable SFA effect relative to an unfamiliar face (i.e., SF–UF).

Findings from this study showed that British Caucasian participants searched more accurately and faster for all faces, regardless of the face identity, compared with Malaysian Chinese participants. In addition, across both race groups, there were no differences in the search accuracy and search time of the own face and friend’s face whereas lower accuracy and longer search times were reported for unfamiliar faces than for those two types of familiar faces. In other words, the SFA effect was absent when the own face was compared to a friend’s face but present when compared with an unfamiliar face, and this finding was observed in both British Caucasian and Malaysian Chinese participants.

Overall, these findings seemed to suggest that (1) one’s own face does not receive preferential processing when compared with another overlearned face (i.e., the friend’s face); (2) both familiar faces showed a processing advantage compared with unfamiliar faces; and (3) the absence of a SFA effect relative to a friend’s face is not modulated by the cultural differences in the self-concepts of participants, not at least in a visual-search paradigm.

### A familiar face advantage rather than a SFA

Tong and Nakayama (1999) reported that the self-face was detected faster among distractor faces compared with an unfamiliar face, even when the self-face was presented in atypical orientations and after hundreds of trials, leading them to suggest a processing advantage for the self-face. However, the authors did not control for possible familiarity effects as the self-face is—due to extensive exposure—a highly overlearned face (Kircher et al., 2001). Hence, a personally familiar face, the face of a friend, was included in this current study to control for such familiarity effects. The lack of differences between the SF and FF in the conjunction with the better detection of these faces compared with an unfamiliar face suggests that the SFA effect may be a result of mere familiarity effect rather than a “self-effect.”

On the other hand, our results are in line with the findings of Devue et al. (2009). With a visual search task where the face identity was deemed irrelevant (i.e., participants were asked to identify a certain mouth configuration), Devue et al. concluded that the own face does not receive attentional prioritisation compared with familiar and unfamiliar faces, such that there was no difference in the searching time between the self and friend’s faces. In addition, Devue et al. showed that the self-face did not receive faster saccade eye movements than other faces. Extending the findings from Tong and Nakayama’s (1999) and Devue et al.’s (2009) studies by including a personally familiar face and making the face identity to be task relevant, we showed that at the level of detection, preferential processing is not restricted only to the own face but also to other personally familiar faces.

One might argue that in the modern era, individuals might see their own face in photographs and videos more often than individuals from previous generations did, and they might be more familiar with their normal-oriented instead of their mirror-oriented face. However, we need to consider that a substantial part of these photographs and videos are still mirror-reversed, as it is the case in *selfies.* More importantly, photographs and videos offer a poor visual experience about the self-face. In fact, a large amount of research has shown that self-face representations are built through the combination of multisensory information, such as visual, tactile, and proprioceptive (for review, see Estudillo & Bindemann, 2017b). In contrast to photographs and videos, self-reflection in a mirror offers this multisensory experience. For example, when one moves the arm in front of the mirror, the reflection provides synchronous dynamic feedback. Finally, we have recently shown similar identification performance and gaze viewing patterns between mirror-reversed and normally oriented instances of the own face (Lee et al., 2022).

Notably, aside from observing a significant SFA effect relative to unfamiliar faces, a processing advantage for the friend’s face compared with an unfamiliar face was also reported. Findings from this study seem to be consistent with the position that there are quantitative differences between the processing of familiar and unfamiliar faces (Bruce et al., 2001; Estudillo, 2012; Gobbini et al., 2013; Ramon et al., 2011; Van Belle et al., 2010). In other words, face processing varies according to face familiarity. For instance, although personally familiar faces and famous faces have a processing advantage over unfamiliar faces, personally familiar faces benefit from a processing advantage compared with famous faces (e.g., Herzmann et al., 2004; Keyes & Zalicks, 2016; but see Wiese et al., 2021). Accordingly, there seems to be a continuum of familiarity within faces that ranges from unfamiliar faces to familiar faces, which includes one’s own face (see Bortolon et al., 2018). Regarding the comparison of the own face, friend’s face, and an unfamiliar face, our findings suggest that there is no preference for the own face over a personally familiar face. Due to extensive exposure, the own face is an overlearned and highly familiar stimuli; hence, it is possible that the processing advantages for the self-face may be attributed to its familiarity rather than from any special “self-effects” (see Lee et al., 2007). In addition, like the own face, the friend’s face may also carry a high emotional load (see Cygan et al., 2014) and they too are encountered often in day-to-day life, and arguably, one may see the friend’s face more often than one may see themselves. Consequently, there might be no difference in the attentional prioritisation to the own face and friend’s face.

Overall, the higher search accuracy and shorter search times for the own face in this current study might be better explained by a familiarity effect. That is, the result of a more robust representation of one’s own face (and friend’s face) due to frequent exposure to one’s own image through the mirror and photos and an extensive experience with highly familiar individuals (Tong & Nakayama, 1999). Likewise, the poorer performance for the unfamiliar faces can also be explained by a less robust representation of unfamiliar faces. In a similar vein, the processing advantage for familiar faces could also be explained by face processing models, such that the person identity nodes and face recognition units process information of familiar faces faster than less familiar faces (Bruce & Young, 1986) due to easier access of stored representation or semantic information (Kircher et al., 2001).

### Discrepancies in task demands

Although our findings suggest that the SFA can be explained in terms of familiarity, other studies have reported evidence of an SFA even when compared with personally familiar faces (e.g., Keyes et al., 2010; Liew et al., 2011; Ma & Han, 2010, 2012; Martini et al., 2015) and famous faces (e.g., Mengya et al., 2013; Miyakoshi et al., 2008; Tacikowski et al., 2011). The lack of consistency across studies may be attributed to the high variability in the design and tasks used by researchers. In their meta-analysis, Bortolon and Raffard (2018) reported that while an SFA was reported for memory (i.e., judging identity) and perception (i.e., identifying head orientation) based tasks, SFA was not reported for attention-based tasks (i.e., simple detection or visual search). Specifically, participants recognised the own face faster compared with other familiar and unfamiliar faces (e.g., Keyes et al., 2010; Liew et al., 2011) but there were no differences between the own, familiar (close others or famous people), and unfamiliar faces in visual search (e.g., Devue et al., 2009; Lee et al., 2007) and face detection tasks (e.g., Cygan et al., 2014; Kotlewska & Nowicka, 2015). The latter finding led Bortolon and Raffard (2018) to suggest that all faces, regardless of identity, are detected at a similar speed in a task involving attentional processes. In line with this view, it is possible that the advantages of self-relevant information (i.e., the self-face) may not affect a prioritisation in the early perceptual stages but rather reflect a prioritisation in later processing stages, such as memory encoding and response selection (e.g., Firestone & Scholl, 2015).

Arguably, it is also conceivable that participants are likely inexperienced at searching for their own face, such that they are asked to search for a small grey-scaled image of their face among an array of distractor faces. In contrast, it is a much more familiar task for participants to search for faces of their close friends. In other words, individuals may be more accustomed to the task of picking out a familiar face in a crowd rather than identifying the own face among an array of different faces (Kircher et al., 2001). Likewise, participants could also be inexperienced in searching for an unfamiliar face among other faces, but due to the own face benefitting from a more robust mental representation, the own face was still searched faster and more accurately than the unfamiliar face.

Hence, one may argue that the lack of SFA relative to a friend’s face in our study may be due to the type of task employed. We would, however, like to highlight that even though our findings showed that there were no differences in the search performance between the own face and friend’s face, there is an advantage in the search time and search accuracy for familiar faces (i.e., self-face and friend’s face) compared with unfamiliar faces. Rather than attributing the lack of SFA relative to a friend’s face to the type of task employed, our findings are in line with the hypothesis that familiar faces are processed faster and more accurately due to a more robust mental representation and further reinforced the proposition that the own face might just be another highly familiar face.

### No cultural modulation effects on SFA

Contradicting our hypothesis and findings from previous studies (e.g., Liew et al., 2011; Sui et al., 2009; Zhang & Zhou, 2019), our findings showed that the search for self-faces was not influenced by the cultural differences in self-concept. Our findings showed that British Caucasian participants searched the self-face faster than Malaysian Chinese participants across both present and absent trials, but British Caucasian participants also searched the friend’s and unfamiliar faces faster (and more accurately) than Malaysian Chinese participants.

We infer that the advantage in the search time for the self-face in British Caucasians compared with Malaysian Chinese cannot be accounted by the cultural differences in self-construal for two reasons. First, our findings showed that British Caucasian participants were overall more accurate and faster than Malaysian Chinese participants when searching for faces, regardless of the identity. Second, our findings from the regression analyses further indicated that the SFA (in terms of search accuracy and search time) relative to an unfamiliar face could not be explained by the cultural differences in self-construal (i.e., operationalised in terms of the scores on the SCS and HCIV questionnaires) of the participants. Race of participants, however, could account for the variability in the search accuracy and search time between the self-face and the unfamiliar face and between the friend’s face and the unfamiliar face. Although previous research has shown cultural differences in the visual search of simply patterns between East Asian and British Caucasian participants (Ueda et al., 2018), with the current data, we cannot determine whether our British Caucasian participants were simply more engaged with the task or whether, compared with Malaysian Chinese, they presented a stronger bias towards faces. Future studies could test this idea by comparing British Caucasian and Malaysian Chinese participants searching for faces and non-face stimuli (e.g., shapes).

## Conclusion

In conclusion, the findings showed in a visual-search paradigm that although there is an SFA relative to unfamiliar faces across both British Caucasian and Malaysian Chinese participants, an SFA was not reported when compared with a friend’s face, and these findings are not modulated by the cultural differences in one’s self-concept. Taken together, this work seemed to suggest that, on a behavioural level at least, the observed SFA is better explained by a familiar face advantage rather than a processing advantage for the self-face. In other words, because we encounter our own face and friend’s face often, we have more robust mental representations of our own face and the face of our friend in comparison to unfamiliar faces whose mental representations are less robust. However, we do not have a more robust representation of our own face when compared with the representative of a friend’s face.
